# Factors Impacting on Development and Implementation of Training Programs for Health Professionals to Deliver Brief Interventions, with a Focus on Programs Developed for Indigenous Clients: A Literature Review

**DOI:** 10.3390/ijerph17031094

**Published:** 2020-02-09

**Authors:** Saji Sebastian, David P. Thomas, Julie Brimblecombe, Vongayi Majoni, Frances C. Cunningham

**Affiliations:** 1 Wellbeing and Preventable Chronic Disease Division, Menzies School of Health Research, Darwin 0810, Northern Territory, Australia; david.thomas@menzies.edu.au (D.P.T.); vongayi.majoni@menzies.edu.au (V.M.); 2 Faculty of Medicine, Monash University, Melbourne 3168, Victoria, Australia; julie.brimblecombe@monash.edu.au; 3 Wellbeing and Preventable Chronic Disease Division, Menzies School of Health Research, Brisbane 4000, Queensland, Australia; frances.cunningham@menzies.edu.au

**Keywords:** brief intervention, brief therapy, CFIR, training program, chronic disease, Indigenous

## Abstract

This paper reviews the literature on evaluations of brief intervention training programs for health professionals which address one or more lifestyle factors of chronic disease to identify factors impacting on development and implementation of programs. A search was conducted of the literature evaluating brief intervention training programs from 2000–2019 in the databases: Medline, CINAHL, Psychinfo, Academic Premier, Science Direct, Ovid (Including EMBASE and Healthstar), Web of Science and Informit. The content analysis and data extraction were aligned to the domains in the Consolidated Framework for Implementation Research (CFIR) to assist in the narrative synthesis. The search identified eight evaluations of programs targeting multiple risk factors, and 17 targeting single risk factors. The behavioural risk factor most commonly addressed was smoking, followed by alcohol and drug use. Programs consisted of face-to-face workshops and/or online or distance learning methods. Facilitators included availability of sustainable funding, adapting the program to suit the organisation’s structural characteristics and adoption of the intervention into routine client care. For Indigenous programs, the use of culturally appropriate images and language, consultation with Indigenous communities, and development of resources specific to the communities targeted were important considerations.

## 1. Introduction

The increasing prevalence of chronic disease is a global issue affecting all countries and populations. Tobacco use, foods high in saturated and trans fats, salt, and sugar (especially in sweetened drinks), physical inactivity, and the harmful consumption of alcohol cause more than two-thirds of all new cases of chronic disease [1]. Whilst there have been significant gains in the management of chronic diseases in recent decades in Australia, they continue to be the leading cause of mortality and morbidity [2]. The exposure to risk factors and the prevalence of chronic disease are not uniformly distributed. For example, Australian Aboriginal and Torres Strait Islander people (referred to as Indigenous Australians in this paper) suffer a higher burden of disease compared to other Australians [3,4]. Indigenous Australians’ poorer general health is reflected in their reduced life expectancy, approximately ten years lower than the general population [5,6].

Preventive efforts at multiple levels of the health care continuum have helped to combat the risk factors. One approach is building the capacity of health workers to deliver brief interventions (BIs) to their clients presenting with such behavioural risk factors. Provision of brief interventions in the primary care setting has been identified as an evidence-based intervention to prevent, detect and manage chronic disease [7,8]. However, the challenges of training health professionals in BI delivery, and the factors that support or impede the provision of BI training programs are not well known. Studies have reported that systemic barriers such as: lack of time of health services and personnel, and lack of policies and systems in place can stifle the uptake of BI training programs [9,10]. This literature review was conducted to inform the evaluation of a brief intervention training program that currently operates in Australia. The Queensland B.strong Program aims to build the capacity of Queensland’s frontline health and community workers to deliver smoking cessation, nutrition and physical activity brief interventions to their Aboriginal and Torres Strait Islander clients [11].

The term ‘Brief Intervention Training’ (BI Training) that is used in this article is widely used, and it means ‘training in the delivery of brief interventions’. This article is a review of literature on programs that train health professionals in delivering brief interventions, with a particular interest in the training programs that are developed for health professionals working with Indigenous populations. This literature review addressed the following question: “What factors impact the development and implementation of training programs for health professionals to deliver brief interventions to their clients, with a focus on programs developed for Indigenous clients?”

## 2. Methods

This review searched electronic databases and other sources from 10 February 2018 to 07 March 2018 initially, and the search was updated on 27 May 2019. In this review we included all types of research studies: case-control, case studies, randomised controlled trials, qualitative and mixed methods studies.

### 2.1. Inclusion and Exclusion Criteria

Articles were included if they were: evaluations or studies of BI training programs that trained health professionals to deliver BIs to modify one or more health behaviours of general and Indigenous populations, and if they were published from 1 January 2000 to the search date. The review considered a range of locations where the training occurred, including hospitals and community clinics. Articles were excluded if they were: evaluations of delivery of BIs rather than of a training program, conducted as part of a university curriculum or on students (as the latter do not shed light on actual practice implementation), not published in the English language, dissertations, discussion pieces, editorials, and opinion pieces, systematic reviews, and meta analyses.

### 2.2. Search Procedures

To locate literature that specifically evaluated BI training programs for health professionals which targeted behavioural risk factors of chronic disease, a set of search terms was developed. Trial searches were conducted before finalising the search strategy. The search terms were grouped as follows: group 1 consisted of: evaluat* OR assess* OR analys* OR measur* apprais*, group 2 consisted of: brief intervention OR behaviour* intervention OR motivational interview* OR counsel* OR intervention OR interview*, group 3 consisted of: train* OR program* OR activity OR deliver* OR support OR research OR study, and group 4 consisted of four sections: (a) smok* OR tobacco OR nicotine OR smok* cessation OR smok* control OR tobacco control, (b) nutrition OR diet OR obesity OR weight, (c) physical activity OR exercise, and (d) alcohol OR grog OR substance OR drug*.

The search was conducted in two phases. Firstly, eight scientific electronic databases, Medline, CINAHL, Psychinfo, Academic Premier, Science Direct, Ovid (Including EMBASE and Healthstar), Web of Science and Informit, were searched. Secondly, to identify any grey literature, a web search was undertaken in the same period in the Cochrane database, and the websites and publications of the following organisations: Australian Institute of Health and Welfare (AIHW), The Lowitja Institute, Australian Indigenous Health Infonet, The National Aboriginal Community Controlled Health Organisation (NACCHO), Menzies School of Health Research, and health departments of Australian federal, state and territory governments. The search for grey literature was mainly limited to Australia due to constraints of time and resources.

### 2.3. Review Processes

The combined searches of all databases and the web search located 14,972 articles, of which 5776 duplicates were removed. The 9196 articles located from the literature search were screened through a three-step process. Firstly, the lead researcher (SS) assessed the articles by examining the title to verify its fit with the inclusion/exclusion criteria. From the screening of 9196 articles, 245 articles were identified to progress to the next step. Secondly, these 245 articles, based on a screening of their titles and abstracts, were checked against the inclusion criteria. A total of 217 articles were selected from this review process.

**Figure 1:**
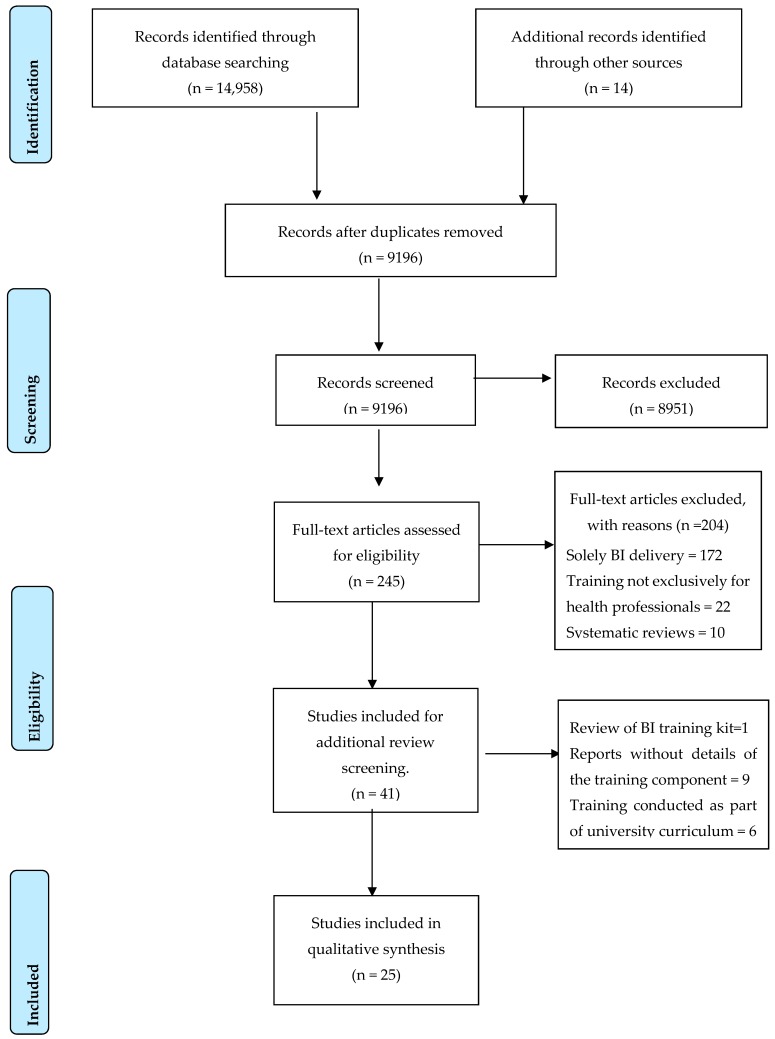
PRISMA flow chart (Inclusion & exclusion criteria).

As the third step, full texts were retrieved for the articles selected in the second step, and the lead researcher (SS) and co-researcher (VM) conducted a review by reading all full text articles. Any disagreements between the lead researcher and the co-researcher on the inclusion/exclusion of articles were referred to a third reviewer (FC). From these processes, 41 articles were identified for inclusion in the literature review. Studies that were reviews of BI training kits, reports of training and development without details of the training component and training conducted as part of a university curriculum or on students were excluded. Sixteen studies were excluded in this process, resulting in the final count of 25 articles for the literature review. The search results are presented in the PRISMA flow diagram (Figure 1).

To synthesise and analyse the literature to identify factors impacting on training program implementation, this review applied the five domains of the Consolidated Framework for Implementation Research (CFIR) [12]. CFIR’s five domains are classified into 26 constructs and 13 subconstructs. The 26 constructs within the five domains of the CFIR are shown below in Table 1.

CFIR, as a conceptual framework, can guide systematic assessment of multilevel implementation contexts to identify factors that might influence intervention implementation and effectiveness [13], and to understand potential barriers and facilitators in preparation for implementing an innovation [12]. The CFIR framework was selected because the domains of the CFIR broadly address key aspects of program implementation, and the framework has been extensively applied in other program evaluations [14,15]. Study implementation aspects were categorised according to the CFIR domains: the characteristics of the intervention, outer setting, inner setting, characteristics of the individuals, and implementation processes. The qualitative evaluation of the B.strong Program has obtained ethics approval from the Human Research Ethics Committee of the Northern Territory Department of Health and Menzies School of Health Research: HREC Reference Number: 2018-3201.

## 3. Results

Nearly half of the 25 retrieved studies (12) were conducted in the United States (US), eight in Australia and three in the United Kingdom. Table 2 displays the key characteristics of the studies included in this review. Most studies/programs (15/25) were conducted solely for research purposes, and not as operational programs. These studies were mainly descriptive. The operational programs, however, provided deeper analysis through measuring changes in trainees’ skills and competence, and included discussions about the contextual factors that impacted on the implementation.

Eleven studies specifically looked at the broader aspects of program implementation by analysing the factors that supported or hindered the processes of training: the program’s fit with the current practices of trainees, policies and protocols in place within health services to support the training program and the use of systems for recording and reporting BI delivery. Three studies evaluated client/patient satisfaction with the brief intervention they received.

Outcome measures in the different papers varied considerably, and they broadly examined the impact on the trainees in the areas of satisfaction with the training and improvements in confidence, motivation, skills and knowledge pre- and post- intervention, cost of the intervention, implementation of brief intervention with clients, trainees’ perceptions of role adequacy and self-efficacy, clients’ satisfaction with the brief intervention provided, reported changes in clients’ engagement in the risk behaviour, access to resources, and exploration of policies and protocols within health services to support the activity. Three studies conducted audits of client clinical charts to determine the quantity and quality of chart recordings of brief interventions [23,31,36]. In one study, reductions in baseline screening test scores for alcohol and smoking were observed in a 3-month follow-up [21]. A high proportion of studies reported on programs that specifically targeted Indigenous populations (n = 4, 16%) and they were all from Australia (50% of the Australian studies). Evaluations of BI training programs targeting Indigenous populations that were available from other countries were not related to assessing change in behavioural risk factors of chronic disease, and hence were not included in this review.

Of the studies identified in this review, there were six programs that had more than 200 trainees (14,762, 1051, 1020, 519, 488 and 441), nine programs had between 51 and 200 trainees, 10 programs had under 50 trainees; while one program did not specify the trainee numbers. The program with the largest number of trainees, the ‘Country-wide distance training for delivery of screening and brief intervention for problematic substance use’ [21] conducted in Brazil trained 14,762 health workers through a distance learning program. The other three large programs were conducted in Australia with the common objective of training health professionals working with Indigenous people to provide smoking cessation advice. The SmokeCheck Program [33] in the state of Queensland trained 441 health workers and the NSW SmokeCheck Program [37] in the state of New South Wales trained 519 health workers. The Quitskills program (2019) implemented in all eight states and territories of Australia [30] trained 1020 health workers.

### 3.1. Factors that Impacted on the Implementation of the Program

The five domains and the 39 constructs and subconstructs of the CFIR were used to categorise the factors that impacted on the implementation of the different studies (Table 3 and Table 4). In conducting the review, we rigorously checked if there were any factors that influenced the implementation of the intervention that did not come under any of the constructs of the CFIR. No factors were identified which could not be classified to one of the CFIR constructs.

#### 3.1.1. The CFIR Domain of Intervention Characteristics

The domain of intervention characteristics relates to the features of the intervention that impact on its adoption and application. These features are classified into eight constructs: Intervention Source, Evidence Strength and Quality, Relative Advantage, Adaptability, Trialability, Complexity, Design Quality and Packaging, and Cost.

A construct of this domain that was most frequently addressed in the different studies was ‘Adaptability’, which signifies the ability of an intervention to be tailored to meet local needs. For example, programs that targeted Indigenous clients developed culturally appropriate resources, and programs that trained a mix of health workers made adjustments to the curriculum to suit the trainee population from different disciplines and cultural backgrounds, and with varying levels of education [21,30,33,37]. The findings of these studies suggest that adaptability of the programs was perceived to have supported their adoption by different health services, and the benefit of such adaptability in the application of the programs was highlighted in the studies.

Programs that used resources developed by educational institutions or universities [19,25,40] were perceived to be of high credibility by the participants. Although many of the programs were supported by government funding, none of the studies reported specifically applying government guidelines in the development of resources. Four training programs incorporated online components, either as standalone or to supplement face-to-face workshops [19,21,26,36]. For the conduct of large-scale programs, particularly those that spanned large geographical areas, web-based programs were found to be beneficial [21] in facilitating learning although they were mainly used as an additional component to face-to-face training [16,19,36].

#### 3.1.2. The domain of Outer Setting

The domain of outer setting incorporates four constructs: patient needs and resources, cosmopolitanism, structural characteristics, and external policies and incentives. The construct that was most often mentioned, in ten studies [18,21,23,27,28,30,31,33,36,37] was ‘Patient Needs and Resources’ that is defined as the extent to which patient needs, as well as barriers and facilitators to meet those needs, are accurately known and prioritized.

The influence of the external policies was evident in the development of some of the Australian programs, which were based on a number of national and state level strategies that broadly advised that any intervention designed to address smoking must be tailored to disadvantaged groups and must encourage behaviour change. The materials and resources used for these programs were perceived to be culturally appropriate to Indigenous populations [30,37].

The effect of external supports in the form of incentives and funding on the maintenance of a program was discussed in a few studies by [9,30,33,37]. The Queensland SmokeCheck Program was funded through the state health department, and funding availability was a major enabler for the NSW SmokeCheck Program. The availability of funding supported the conduct of training at multiple sites and also covered the costs of travel [37]. While the funding for the SmokeCheck Programs was not continued from the early 2010s, the South Australian Cancer Council’s Quitskills Program [30] has had continued funding from the Australian Government. The study by Carneiro et al. [21] on another large program, the ‘Country-wide distance learning program’ in Brazil, does not explicitly state the funding source, but the support of the government is implied. This program developed a significant quantity of course material that could be disseminated through online platforms. In summary, the sustainability of funding and the extent to which patient needs are addressed are important considerations in the development and implementation of BI training programs.

#### 3.1.3. The Domain of Inner Setting

The domain of Inner Setting includes five constructs: Structural Characteristics, Networks and Communications, Culture, Implementation Climate, and Readiness for Implementation. For operational programs conducted over large geographical areas, distance was a major structural characteristic that impacted on the implementation. In the SBIRT (screening, brief intervention, referral and treatment) training in the state of New Mexico in the United States [9], two clinical supervisors were engaged to travel around the state of New Mexico, to train behavioural health counsellors. For the Brazilian distance learning program in ‘Screening and BI’ [21], the number of professionals proposed to be trained in the program was large, and most sites were in small towns, distant from main health centres. Consequently, web-based and distance learning program resources were used, which suited the structural characteristics of the multiple organisations that were spread across the country. However, as these methods were new to most participants, the project employed strategies such as: constant telephone support, online discussion forums and use of call centres to support participants.

Another large operational program, the Queensland Smokecheck program [33], was conducted through one day face-to-face workshops in 31 locations covering urban, regional and remote areas of the state of Queensland in Australia. The NSW Smokecheck program [37] consisted of participation in a one-day training workshop, conducted in 46 locations for the health professionals, and another 17 workshops for managers, spread over the entire state of NSW in Australia. This project collaborated with health services so that they were involved in the organisation of training workshops. The role of Aboriginal Health Workers as mediators between service providers and communities was acknowledged in this training program [37].

The construct ‘Readiness for implementation’ was referred to eight times. This construct consists of three sub-constructs: Leadership Engagement, Available Resources, and Access to Knowledge and Information. Some of the challenges experienced in the recruitment of training participants for different programs, as reported, for example, in the evaluation of the NSW SmokeCheck Program, were: small staff numbers for some organisations; some organisations did not feel they fitted in with the program; the reluctance on the part of managers and senior health professionals to attend the program; and the lack of managerial support for the implementation of the program [37]. Barriers to implementation of the programs (post training) included differing perceptions of managers about managing the intervention, particularly adoption of the intervention into routine client care, gaps in health professionals’ knowledge and skills impacting on their ability to deliver brief interventions, lack of clear instructions, and a single framework to follow [37]. Carneiro et al. (2017) observed that lack of time, infrastructure or support from service managers, as well as competition with other work activities limited the availability of many professionals to participate in the study processes.

#### 3.1.4. The Domain of Characteristics of Individuals

Out of all five of the CFIR domains, the influence of the characteristics of the trainees on the implementation of the programs has been the least evaluated area. This domain consists of five constructs: Knowledge and Beliefs about the Intervention, Self-efficacy, Individual Stage of Change, Individual Identification with Organization, and Other Personal Attributes. Several programs trained multiple health professionals: nurses, health workers, general practitioners, specialist doctors, and alcohol and community workers. However, none of the studies evaluated how the program’s impact varied according to the professional or personal characteristics of the participants.

#### 3.1.5. The Domain of Implementation Processes

This domain consists of the following constructs: Planning, Engaging, Executing, and Reflecting and Evaluating. The construct of ‘Planning’ was referred to seven times and ‘Engaging’ was referred to five times. The study by Gonzales et al. [9] describes the benefit of utilising the services of a non-profit organisation (SDCCHP), as they were able to work directly with a network of service providers across the state with minimal bureaucratic obstacles, acting as an organisational champion. However, there were no studies in this review that specifically examined the use of individual opinion leaders or evaluated the impact of formally or informally appointed champions other than brief mentions in two studies [17,38]. The study by Harris et al. (2005) [28] notes the importance of the establishment of local planning for the successful implementation of the program. However, they also state that due to this study partnership not being at the most senior levels of the local area health services, the planning of referral pathways in some areas was not as effective as in others [28]. The incorporation of a training session for managers was adopted by a number of interventions in this review on the presumption that implementation, as described above, is influenced by senior staff and systems [27,37].

## 4. Discussion

This review identified that the evaluations of large operational programs such as the Queensland and NSW Smokecheck Programs, the South Australian Cancer Council’s Quitskills Program, Brazil’s Country-wide distance training program and the New Mexico SBIRT project had the potential to probe into the multiple factors that impacted on the implementation of these programs [9,21,30,33,37]. These evaluations and two small operational studies [18,27], and three non-operational studies [28,31,38] provided insights into development of their programs and their resources, and how collaborations were entered with other organisations. They also discussed some of the barriers and facilitators to implementation. The review has shown the importance of: local planning for the successful implementation of the program, ensuring that program partnerships are at the most senior levels of the local area health services, the presence of an opinion leader or a champion, clear instructions and a common framework, adoption of the intervention into routine client care, ensuring sustainability of funding, involvement of reputed organisations in the development of the programs and flexible training methods such as face-to face training and distance learning methods including online learning to meet the characteristics of the organisations.

We know from previous studies that the characteristics of the ‘Inner Setting’ significantly influence implementation efforts. Although professional training in the delivery of brief interventions is usually delivered by external providers, its implementation requires active collaboration with different health services. The size and spread of the health service, the structure and duration of the training program, the materials and resources developed, and the program’s requirement to cater for a mix of health professionals are important considerations in its implementation [41,42]. We also know that systemic barriers of lack of time of health services and personnel, competition with other pressing healthcare needs, and lack of policies and systems in place within the participating organisations are barriers to implementing BI training programs [10,43]. Attitudes towards the behaviour targeted, lack of structural and organisational support, unclear role definition as to responsibility in addressing the behaviour with clients, and fears of damaging professional/ client relationships are also known to be barriers to intervention implementation [42,43].

This review too, based on the number of references to the different CFIR domains, confirmed that the characteristics of the health service (in CFIR terms, the inner setting) play a significant role in the implementation of the program. Besides establishing partnerships at senior levels with the participating organisations from the start, the importance of extending the program or tailoring the training to suit the managers and supervisors of the organisations was identified as helping to ensure better adoption by the health services [37]. This review also touches on the sustainability of the skills gained by health workers through BI training. To address this issue, the review has identified efforts such as: flexible ongoing training methods, in-person site visits, training materials designed specifically for the unit settings, monthly calls or in-person booster sessions to enhance ongoing implementation efforts [39]. For programs that are developed for Indigenous populations, the review affirms the importance of addressing specific client needs and consultation with Indigenous communities [33,37]. Besides considering the characteristics of the client population in the development of the course content and curriculum, it is also important to tailor the course to each community as much as is practically possible [30,33].

In the area of delivery of brief interventions by health professionals to their clients, the availability of resources (learning materials, assessment tools and referral guides) and how they were managed within the organisation, and the program’s fit with the structural characteristics of the organisation were important [39]. BI delivery needs to be developed as a systems-based practice, with BI and referral to treatment services ideally being active processes.

In addition, a review of previously conducted systematic reviews highlighted similar themes and identified the importance of follow-up training sessions and/or training events spread over a longer time period [44]. Whilst the use of objective observational tools for evaluating the fidelity and quality of brief interventions is identified, there is also consensus that formal learning is only one aspect of learning BI skills and that informal learning that occurs in everyday counselling in clinical practice is equally important. The need for further qualitative research was noted in several studies [41,43]. Studies also identified the association between implementation effectiveness (material utilisation, screening, and BI rates) and the intensity of the implementation effort, i.e., the amount of training and/or support provided [42].

### 4.1. Strengths and Limitations

The application of the CFIR in this review has been highly beneficial in guiding the assessment of multilevel implementation contexts to locate factors that influenced implementation and effectiveness of the interventions, particularly in identifying the non-explicit descriptions of barriers and facilitators from the different articles. The studies included in this review differed significantly in terms of the settings, the size and nature of trainee populations, modes of interventions, program content, and outcome measures, and thus provided a comprehensive picture of the intervention environment.

However, most studies extracted in this review were about small programs with few participants, delivered across few sites (usually one site), with a very short period of operation, and usually targeting one health behaviour, and hence conducted little evaluation to gain insights into the factors that impacted on the implementation of the programs. Similarly, although BI programs for multiple health behaviours were reviewed in eight studies, none of the studies specifically identified or described the challenges of conducting multi-behaviour change training or implementation of BIs for multiple risk factors. Whilst key factors that impact on the implementation of brief intervention training programs for the general population were well identified, there was limited information on factors specific to the Indigenous populations. Only a small number of broad factors such as: consulting the communities prior to development of the program, tailoring the program to match the participant population and ensuring the cultural suitability of the programs and resources were identified. Limited research has examined the most effective methods for delivery of BI training to Indigenous health workers who work with Indigenous clients. Similarly lacking is an understanding of the extent of decline in the skills and knowledge that trainees acquired from a BI training program, although two studies reported that participants felt increasingly less legitimate and confident as time elapsed [22,33]. We also have little understanding of the nature and frequency of refresher or ongoing programs to address such gaps. The funding issues associated with refresher programs and the practical challenges of implementing them have also not been covered.

### 4.2. Implications for Future BI Training

The Final Report on NSW SmokeCheck stated that ‘the results point to the need for organisational development on the part of health services to ensure that smoking cessation becomes a standard component of Aboriginal Health Workers’ and other health professionals’ clinical and health promotion roles. The report points to the need for health services to provide organisational support to trainees, highlighting the role of health services’ managerial staff in supporting the implementation of BI training programs. This suggests the need for all future BI training programs to incorporate specific training components for managers. The health practitioners’ perceptions about their knowledge and their right to work in the identified area(s) are important in the ongoing support for the program, and the resultant delivery of BIs. Hence, the design of the programs needs to consider the trainees’ fit with the program: their educational and professional backgrounds and build on their role legitimacy and adequacy. This is particularly important consideration for managers, as the responsibility for preventive care is often perceived to be on front line health professionals rather than on the whole health service.

To increase the accessibility of the program to health workers located in rural and remote locations, the need for the availability and sustainability of funding is highlighted. It is also important to consider alternate modes of delivering the training such as web-based modules and distance learning. Gaps in health professionals’ knowledge and skills continue to exist even after participation in the training program, and erosion in motivation and skills is likely to happen over time, impacting on their ability to deliver brief interventions. To address these concerns and to address the high turnover of staff members, routine repeat courses and refresher programs suitable to the trainee population need to be available. In the context of BI delivery for Indigenous clients, the role of Aboriginal Health Workers as mediators between service providers and communities needs to be acknowledged in the training program [37]. Whilst it is important to develop culturally appropriate resources and learning materials, it is also important to tailor the course to each community as much as is practically possible, particularly to be inclusive of regional cultural and language differences.

## 5. Conclusions

The identification of common contextual factors that impacted on the implementation of the program has been difficult due to the varied nature of the studies. However, the descriptions of the development and implementation of the programs do provide some information on the factors that favoured or hindered the different processes. The most commonly discussed CFIR domain in the different evaluations was the influence of the inner setting or the characteristics of the participating organisations. The application of the CFIR has been particularly beneficial in identifying and teasing out the not so obvious descriptions of the contextual factors from the different articles. A highlight of this review is the generation of the knowledge that the literature on BI training programs for health professionals is generally deficient in understanding the complex interplay of contextual factors that impact on the implementation of the programs.

Key messages from the review for the development and implementation of future BI training programs include: ensuring supports from management and engagement of workers from the start and incorporating the intervention as a standard component of health professionals’ roles. The availability of ongoing funding and adapting the program to meet the structural characteristics of the organisations are also important. Subsequent to the conduct of the training program, ongoing implementation of the intervention needs to be incorporated into routine practice aligning with the operational procedures of the organisation. This review highlights that healthcare providers can attain brief motivational interview knowledge, skills and confidence relatively quickly and these can be sustained over time, however, further research is needed to determine if BI skills are long lasting. Finally, to comprehensively understand the factors that impact on the implementation of BI training programs, conduct of in-depth qualitative studies to evaluate specific training features and methods that work best is recommended. It is expected that the results of such a study would provide deeper insights into facilitators and barriers of organisational implementation of BI training programs and its ongoing delivery.

## Figures and Tables

**Table 1:** Consolidated Framework for Implementation Research (CFIR) Domains and Constructs.

Intervention Characteristics	Inner Setting	Outer Setting	Characteristics of Individuals	Process
Intervention source Evidence strength and quality Relative advantage Adaptability Trialability Complexity Design quality Cost	Structural characteristics Networks and communications Culture Implementation climate Readiness for implementation	Patient needs and resources Cosmopolitanism Peer pressure External policies and incentives	Knowledge and beliefs about the intervention Self-efficacy Individual state of change Individual identification with organization Other personal attributes	Planning Engaging Executing Reflecting and evaluating

**Table 2:** Characteristics of included studies.

Study Reference and Year	Program Characteristics					Study Characteristics	
	Country	Trainee Population	Trainee Numbers (N)	Health Behaviour Addressed	Type of Program	Study Sample	Outcome Measures
Akers L, et al. 2006 [16]	USA	Dental Hygienists	1051	Smoking	Research	n = 1051	Baseline and post-intervention ‘smokeless tobacco-related activities’ with patients. Intervention costs.
Barta, SK. 2002 [17]	USA	Nurses	15	Smoking	Research	n = 15	Self-efficacy and frequency of conducting brief smoking cessation interventions.
Boucek et al. 2019 [18]	USA	Nursing staff	65	Alcohol	Operational	Nursing staff (trainees) = 55 Aged care Residents (clients) = 40	Knowledge of Screening, Brief Intervention, and Referral to Treatment Services (SBIRT) and attitudes toward patients with alcohol use problems. Implementation rates of SBIRT post-training. Resident alcohol consumption.
Butler CC, et al. 2013 [19]	Wales, UK.	Nurses and doctors	27 general practices	Smoking, Nutrition, Alcohol and Physical Activity	Research	Intervention = 831 patients. Control group= 996 patients.	Proportion of patients who reported making beneficial changes in at least one of the four risky behaviours at three months.
Cantor SB, et al. 2015 [20]	USA	Physicians and pharmacists	170	Smoking	Research	170 physicians and pharmacists 888 participants (patients)	Short-term outcomes: Cost per quit. Long-term outcomes: Cost per quality-adjusted life-year (QALY).
Carneiro and Souza- Formigoni. 2017 [21]	Brazil	Health professionals and social workers in the public service.	14,762	Alcohol, Tobacco and Other Drugs	Operational	2420 Health Professionals for the 1^st^ phase 25 Health Professionals for the 2^nd^ phase No. patients = 79	Motivation to deliver SBIs. Support from workplace managers. Perceptions about qualifications to perform SBI. The characteristics of the brief intervention delivered by the health workers. Quality of the SBI delivery. Patients’ level of satisfaction with the SBI.
Christie G. et al. 2013 [22]	New Zealand	Alcohol and Other Drug (AOD) Workers	37	Alcohol and Drugs	Research	T1: n = 37 T2: n = 32 T3: n = 15. Three focus group sessions	Attitudes of workers regarding AOD assessment and brief interventions. Motivation, role adequacy, task specific self-esteem, role legitimacy and work satisfaction.
Clifford A, et al. 2013 [23]	Australia	Aboriginal Community Controlled Health Services (ACCHS) staff	4 ACCHS	Alcohol	Research	100% of clients eligible in 2 ACCHSs (numbers not provided). n = 360 and n = 15 respectively for the other two ACCHSs.	Frequency and quantity of alcohol consumption, and frequency of heavy drinking. Actions or advice (brief intervention) recorded for at-risk clients in the client data file. The proportion of eligible clients, pre- versus post-intervention with: Alcohol screen information, Complete alcohol screen, Heavy drinking screen, Valid alcohol screen, and Brief intervention.
Coogle CL and Owens MG. 2015 [24]	USA	Multiple healthcare practitioners	93	Alcohol (Elderly Population)	Research	n = 93	Commitment (intention to use the learned approaches in professional practice). Intention to recommend training. Level of comfort (to use the technique in professional practice).
Daws C, et al. 2013 [25]	Australia	AOD workers	56 staff from three services	Smoking	Research	56 participants (28 in the intervention group and 28 in the control group)	Individual—the perception of role legitimacy (RL) and role adequacy (RA), and attitudes towards responding to smoking issues. Team—work team dynamics: team culture, workload pressure, team communication, and morale. Workplace—factors in the working environment that may impact on work practice. Organisational—factors that impact on the organisation as a whole. Post-training section: relevance and outcomes of the training.
Edwards EJ, et al. 2015 [26]	Australia	Multiple healthcare professionals	163	Nutrition and Physical Activity	Research	Between 101 and 151 participants over different time periods.	Participant’s knowledge of brief MI and confidence with health behaviour change counselling—Pre, Post, 3-Month Follow-up and 6-Month Follow-up.
Fitzgerald N, et al. 2015 [27]	UK	Multiple health professionals	89	Alcohol	Operational	n = 15	Perceived need for and approaches to Alcohol Brief Intervention delivery. Compatibility of Alcohol Brief Interventions with current practice.
Gonzales A, et al. 2012 [9]	USA	Behavioural Health Counsellors and Supervisors.	28	AOD	Operational	Screened, n = 53, 238 adults. Eligible for brief intervention, n = 6,360.	Substance use, living conditions, employment, criminal justice, mental and physical health, and social connectedness.
Harris et al. 2005 [28]	Australia	General practitioners	21	Smoking, Nutrition, Alcohol and Physical Activity (SNAP)	Research	21 General Practitioner (GP) Clinics	Capacity for implementing SNAP at the practice level. Skill and knowledge in assessing and offering interventions for patients with SNAP risk factors. Assessing readiness to change. Conducting motivational interviewing and patient education. Management of patients who were smokers, overweight or at-risk drinkers.
Kerr S, et al. 2011 [29]	Scotland	Nurses and allied health professionals	73	Smoking	Research	T1 = 28 and 29 T2 = 27 and 27 T3 = 27 and 25. Qualitative: (n = 8).	Participants’ views of satisfaction with the training. Effectiveness of the training post training: assessing knowledge, therapeutic attitudes and reported practice.
Martin et al. 2019 [30]	Australia	Health professionals	1020	Smoking	Operational	Pre-workshop survey n = 787. Post-workshop survey n = 765. Four six-week follow-up survey n = 416.	Participants’ level of agreement on their knowledge, skills and confidence to address tobacco BIs. The strengths and weaknesses of the training, and any other course feedback. Use of skills acquired through the course, tobacco-related changes made at an organisational level, and participant views of the cultural relevance of the program.
Olaiya O, et al. 2015 [31]	USA	Staff at Women, Infants and Children (WIC) clinics.	38 clinics	Smoking	Research	Exact number of workers trained is not given. 71,526 pregnant smokers were recruited.	Quitting smoking. Women who reported smoking no cigarettes during the last 3 months of pregnancy were categorized as having quit.
Payne, et al.2014 [32]	USA	Multiple health professionals	488	Tobacco use	Research	n = 488	Practice behaviour, self-efficacy and attitude ratings of trainees at pre-training, post training and 6-month follow-up.
Queensland Health Smokecheck. 2007 [33]	Australia	Health workers	441	Smoking	Operational	T1 = 217 T2 = 133 T3 = 87 Indigenous clients (n = 143)	Use of brief interventions to reduce tobacco smoking, preparedness to conduct brief intervention, skills indDelivering brief intervention, role legitimacy, confidence in delivering a brief intervention, importance of reducing communitylLevels of smoking, and feasibility of SmokeCheck brief intervention and program uptake.
Rosenthal et al. 2018 [34]	USA	Nurses	48	Alcohol and Substance use	Operational	Online training, n = 48 In-person classroom: n = 28	Application of SBIRT (simulation score). Use of open-ended questions, reflection, conversations that referenced outside support (referral to treatment) and use of eye contact and appropriate body language.
Schwindt et al. 2019 [35]	USA	Nurses	12	Substance use	Research	n = 12	The degree to which trainees agreed with the overall quality of the training, and their satisfaction with the training materials, facilities, and presenters. Three open-ended questions to solicit additional comments.
Simerson D, and Hackbarth D. 2017 [36]	USA	Nurses	74	Smoking	Operational	74 nurses participated in the BI training and the post-training survey. Data were collected on 7,465 emergency visits.	Knowledge about brief smoking-cessation intervention methods.
University of Sydney. 2010 [37]	Australia	Aboriginal Health Workers and health professionals	519	Smoking	Operational	n = 499	Health professionals’ knowledge, confidence, perceptions, motivation, skills and actions to deliver smoking cessation brief interventions to Aboriginal clients. Policies and protocols in place within health services to support this activity. Access to quit smoking resources, and access to culturally appropriate quit smoking resources.
Whitty, et al. 2016 [38]	Australia	Doctors, nurses and allied health professionals	59	Alcohol	Research	Ten key informants. 58 health professionals.	Knowledge of alcohol screening, brief alcohol interventions and relevant referral services. Confidence in asking patients about drinking habits, talking about addressing alcohol issues and arranging patient referrals. Feedback on the conduct of the sessions, usefulness of the resources and cross-cultural partnerships.
Zimmermann et al. 2018 [39]	USA	Social Workers	Not provided	Alcohol	Operational	All social workers (numbers not provided)	Identification of patients. Communication and program feedback

**Table 3:** Detailed references of CFIR Domains and Constructs in the reviewed studies.

Study Reference and year	Country	CFIR Constructs Addressed (Domains: I. Intervention Characteristics, II. Outer Setting, III. Inner Setting, IV. Characteristics of Individuals, V. Process)
Akers L, et al. 2006 [16]	USA	Planning (V): Two different study modules developed.
Barta SK. 2002 [17]	USA	Nil
Boucek L, et al. 2019 [18]	USA	Intervention Source and Evidence Strength and Quality (I): Program is based on the SBIRT training manual developed at the University of Pittsburgh School of Nursing. Patient Needs and Resources (II): 40% of older adults drink alcohol. Leadership Engagement (III): Nursing manager requested the development of the program.
Butler CC, et al. 2013 [19]	Wales, UK.	Culture and Implementation Climate (III): The design of randomising by cluster at the level of general practice.
Cantor SB, et al. 2015 [20]	USA	Nil
Carneiro and Souza- Formigoni. 2017 [21]	Brazil	Adaptability (I): Intervention was designed to meet the geographic spread of a large number of health workers. Patient Needs and Resources (II): The little knowledge of most of the health professionals and social workers on substance-related problems was acknowledged and addressed in the development of the program. Planning and Engaging (V): The program was developed comprehensively and well engaged with the participants through multiple mediums.
Christie G. et al. 2013 [22]	New Zealand	Adaptability (I): Flexible intervention and adapting to meet needs of specific client groups. Leadership Engagement (III): Support services being readily available for referring patients to. Available Resources (III): Having available quick and easy screening questionnaires and intervention techniques.
Clifford A, et al. 2013 [23]	Australia	Adaptability (I): Tailored outreach support. Patient Needs and Resources (III): Prior research into factors influencing alcohol SBI. Reflecting and Evaluating (V): Pre- and post-assessment of alcohol information recorded in the electronic patient information systems.
Coogle CL and Owens MG. 2015 [24]	USA	Adaptability (I): The training sessions varied in length. Other personal attributes (IV): Professional Group Differences in Outcome Measures were evaluated.
Daws C, et al. 2013 [25]	Australia	Leadership Engagement (III): Introducing the training as part of a workforce development opportunity.
Edwards EJ, et al. 2015 [26]	Australia	Nil.
Fitzgerald N, et al. 2015	UK	Patient Needs and Resources (II): Some of the client needs and the barriers to introducing interventions have been discussed – and supported the development of the program.
Gonzales A, et al. 2012 [9]	USA	Cosmopolitism (II): Conducted through a locally based, non-profit organization. Planning and Engaging (V): Collaborated with 8 community health centres.
Harris et al. 2005 [28]	Australia	Patient Needs and Resources (II): Needs assessment conducted. External Policies and Incentives (II): NSW Health funded a feasibility study on the SNAP approach to behavioural risk factor management. Leadership Engagement and Access to knowledge and information (III): The program supported development of resources and engaged with multiple organisations.
Kerr S, et al. 2011 [29]	Scotland	Adaptability (I): The program was designed to address the pessimistic attitudes among the clients, and trainees about smoking cessation among older adults.
Martin et al. 2019 [30]	Australia	Intervention Source and Evidence Strength and Quality (I): Facilitators from Cancer Council SA have delivered the course. Adaptability (I): Course delivered over various times in multiple locations. Patient Needs and Resources (II): Tobacco use accounts for high burden of disease for Aboriginal and Torres Strait Islander people. External Policies and Incentives (II): Funded by the Australian Government as part of the Tackling Indigenous Smoking Program. Culture (III): Some organisations may have made it compulsory for their staff to attend the program.
Olaiya O, et al. 2015 [31]	USA	Patient Needs and Resources, Cosmopolitism and External Policies and Incentives (II): Recognizing the need to improve perinatal smoking cessation, the Ohio Department of Health trained select WIC clinics—who reach a large proportion of low-income women during the perinatal period. Structural Characteristics and Networks and Communications (III): ODH staff provided technical assistance to help clinics integrate the steps of the 5As into clinic procedures and conducted periodic chart reviews.
Payne, et al. 2014 [32]	USA	Evidence Strength and Quality (I): Training delivered by doctoral level psychologists affiliated with the University of Mississippi Medical Center. Two trainers were present at each training. Organizational Incentives and Rewards (III): Continuing education credit.
Queensland Health Smokecheck. 2007 [33]	Australia	Structural Characteristics, Networks and Communications and Implementation Climate (III): Needs identified, the intervention was spread around a whole state engaging multiple organisations, national priorities acknowledged. Tension for Change and Access to knowledge and information (III): Consensus among stakeholders about the size of the problem – smoking, Resources and study materials specific to the program were developed. Planning, Engaging and Executing (V): The program was planned and executed as designed.
Rosenthal et al. 2018 [34]	USA	Evidence Strength and Quality (I): Educational needs assessment was conducted with the RNs on the project unit. Adaptability (I): Sessions were offered at different times to boost attendance. Relative Advantage (I): Other groups of providers also received information and briefings regarding the entire project.
Schwindt et al. 2019 [35]	USA	Evidence Strength and Quality (I): Trainings were led by qualified professionals. Adaptability (I): Site coordinators who were not able to attend a face-to-face training completed an online training module. Formally Appointed Internal Implementation Leaders (V): One RN was selected as study site coordinator who was best positioned to lead study activities at that site.
Simerson D, and Hackbarth D. 2017 [36]	USA	Intervention Source, Evidence Strength and Quality and Trialability (I): The training module was developed using input from the needs-assessment survey from among the participants. Patient Needs and Resources (II): It was identified that no record of the delivery existed of any type of smoking-cessation intervention.
University of Sydney. 2010 [37]	Australia	Patient Needs and Resources, Cosmopolitism and External Policies and Incentives (II): The program was developed in response to the concerns of NSW Health about the high rates of smoking in NSW Aboriginal communities, the intervention engaged multiple organisations. Tension for Change and Access to knowledge and information (III): The size of the problem is well discussed and documented. Resources and study materials specific to the program were developed. Planning, Engaging and Executing (V): The program was implemented through multiple organisations in the whole of NSW.
Whitty, et al. 2016 [38]	Australia	Cosmoplitism (I): Collaboration with key stakeholders and service providers. Leadership Engagement (III): Training and adherence checks by superiors. Access to knowledge and information (III): Brief screening tools. Champions (V): Presence of a clinical champion. Planning (V): Small in-hospital training session.
Zimmermann et al. 2018 [39]	USA	Evidence Strength and Quality (I): Trainees attended a New York State Department of Health SBIRT training session. Individual Identification with Organization (IV): The importance of social workers in the treatment process. Planning (V): The implementation project began by assembling a multidisciplinary team.

**Table 4:** CFIR Domains referred to in studies.

Domains	No. of Times Referred to
I. Intervention characteristics	21
II. Outer Setting	15
III. Inner Setting	23
IV. Characteristics of Individuals	02
V. Process	15
Total	76
